# Simulations of the synthesis of boron-nitride nanostructures in a hot, high pressure gas volume†
†Electronic supplementary information (ESI) available. See DOI: 10.1039/c8sc00667a


**DOI:** 10.1039/c8sc00667a

**Published:** 2018-03-19

**Authors:** Predrag S. Krstic, Longtao Han, Stephan Irle, Hiromi Nakai

**Affiliations:** a Institute for Advanced Computational Science , Stony Brook University , Stony Brook , NY 11794-5250 , USA . Email: predrag.krstic@stonybrook.edu; b Department of Materials Science and Chemical Engineering , Stony Brook University , Stony Brook , NY 11794-2275 , USA; c Computational Sciences & Engineering Division , Oak Ridge National Laboratory , Oak Ridge TN , 37831-6493 , USA; d Department of Chemistry and Biochemistry , School of Advanced Science and Engineering , Waseda University , Tokyo 169-8555 , Japan; e Waseda Research Institute for Science and Engineering , Waseda University , Tokyo 169-8555 , Japan

## Abstract

We performed nanosecond timescale computer simulations of clusterization and agglomeration processes of boron nitride (BN) nanostructures in hot, high pressure gas, starting from eleven different atomic and molecular precursor systems containing boron, nitrogen and hydrogen at various temperatures from 1500 to 6000 K. The synthesized BN nanostructures self-assemble in the form of cages, flakes, and tubes as well as amorphous structures. The simulations facilitate the analysis of chemical dynamics and we are able to predict the optimal conditions concerning temperature and chemical precursor composition for controlling the synthesis process in a high temperature gas volume, at high pressure. We identify the optimal precursor/temperature choices that lead to the nanostructures of highest quality with the highest rate of synthesis, using a novel parameter of the quality of the synthesis (PQS). Two distinct mechanisms of BN nanotube growth were found, neither of them based on the root-growth process. The simulations were performed using quantum-classical molecular dynamics (QCMD) based on the density-functional tight-binding (DFTB) quantum mechanics in conjunction with a divide-and-conquer (DC) linear scaling algorithm, as implemented in the DC-DFTB-K code, enabling the study of systems as large as 1300 atoms in canonical NVT ensembles for 1 ns time.

## Abstract 2

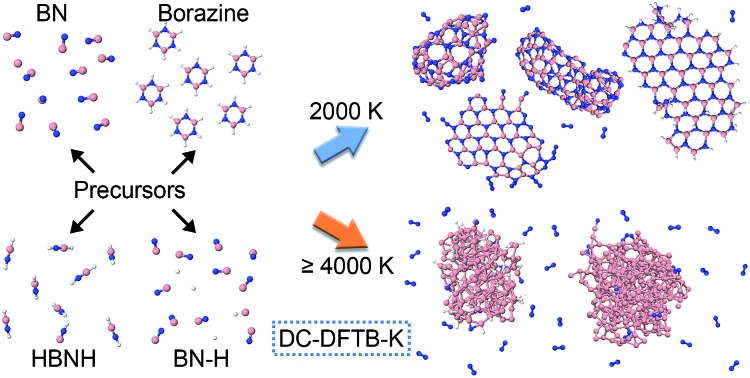
Quantum-classical molecular dynamics reveals optimal molecular precursors and temperatures for synthesis of boron-nitride nanostructures.

## 1. Introduction

Boron nitride, BN, is a compound isoelectronic with elemental carbon and can appear in similarly rich crystalline phases, including fullerene-like (0D), nanotubes (1D, BNNTs), hexagonal graphene-like (2D, h-BN) and diamond-like (3D, cubic c-BN).^1^ Different from carbon, most of these phases do not naturally occur but must be synthesized from their elements.^2^ Also different from carbon, BN nanostructures (BNNSs) are remarkably chemically stable^3^ and heat-resistant.^4^ Furthermore, all defect-free BNNSs exhibit a wide band gap^5^,^6^ and are thus of great interest for a wide variety of technological applications in cases where electrically insulating properties are desirable, ranging from optoelectronics to mechanics.^6^,^7^

Despite the close structural relationship with carbon nanostructures, the factors governing the synthesis and production of BNNSs are different from their carbon analogues,^8^,^9^ owing to the B–N chemistry that involves both ionic and covalent bonding, and the need for B–N element alternation in any thermodynamically stable BN structure. BNNTs, which are harder to synthesize than carbon nanotubes (CNTs),^10^ have been grown by arc discharge,^11^ laser ablation,^12^ substitution reactions from CNTs,^13^ ball-milling,^14^ chemical vapor deposition (CVD),^11^ boron oxide CVD (BOCVD)/floating zone method,^15^ and others.^16^ A significant difference between the production of a single-walled BNNT (SWBNNT) and their carbon SWCNT analogues is that no metal or other catalyst is needed for the BNNT synthesis.^17^ In the case of SWCNT synthesis, a transition metal catalyst is usually required to drive the production of single-walled nanotube structures, unless CVD synthesis is performed under special conditions on a substrate^18^ or from a seed template.^19^ Nevertheless, similar to the case of carbon nanostructures,^20^ the nucleation and growth mechanism of 0D nano-cocoons and nanocages, 1D BNNTs, and 2D h-BN flakes, is still under debate.^21^ Also in analogy to the discussion of the CNT growth mechanism, a root-growth model was postulated for BNNTs^22^ and is repeatedly cited in the literature as the prevailing mechanism of BNNT nucleation and growth.^23^ In the BNNT root-growth model, the nanotube extrudes from a boron cluster, which catalyses insertion of boron and nitrogen atoms at the cluster–tube interface. Our recent theoretical simulations, based on mixed quantum-classical molecular dynamics (QCMD),‡
‡In our QCMD methodology, within the framework of the Born–Oppenheimer approximation we solve the quantum-mechanical (QM) eigenvalue problem for electronic motion with fixed positions of nuclei at each time step (1 fs) of the classical molecular dynamics. The atoms are then released to move classically, driven by the forces calculated from the QM solution for the duration of one step, after which the atom positions are again fixed, and the QM solution for electrons updated. One may argue that the label Born–Oppenheimer MD (BOMD) better conveys that nuclei are treated classically while the electrons are treated quantum-mechanically. indicate that highly organized BNNS such as BN cages and BNNTs can self-assemble simply from BN or BN-containing molecules without the presence of a pre-existing catalyst nanoparticle,^24^ as well as by exposure of boron clusters to a nitrogen atmosphere.^24^–^26^ In both cases, high temperature conditions of around 2000 K are shown to be crucial in the process, enabling fast diffusion of N, B or BN species to an adequate lowest energy position in the nanolattice, usually towards structural defects and the open or closed ends of the tubes. We further reported that the presence of hydrogen in the plasma could also play a decisive role by driving the change of sp-hybridization of B and N atoms from trivalent sp^2^ to tetravalent sp^3^,^25^ in agreement with experimental observation.^27^ The simulations suggest that increasing the hydrogen content in the plasma induces a phase change from h-BN to c-BN, and influences the rate of production of multi-walled cages and BNNTs.^25^ It is hypothesized in the literature that hydrogen in nitrogen plasma is necessary to prevent the recombination of nitrogen atoms to non-reactive dinitrogen molecules.^8^,^23^

The conditions of the high temperature and high flux of the precursors in the described processes can be reached in plasmas at high pressure, like plasma torch^21^ and plasma arc,^11^,^28^ which are the applications we primarily consider in the development of the present theory and simulations. The main objective of this work is to investigate the roles of the precursor content, temperature and time for clustering and agglomeration of nanoparticles, in an atmosphere of boron, nitrogen, and where applicable, hydrogen atoms and/or small chemical compounds, within a temperature range from 1500 to 6000 K. This simulation design differs from our previous studies where nanoparticles were already present from the start of the simulations.^24^,^25^ Due to the use of the NVT canonical ensemble in our simulations, the pressure changes with temperature, and it also changes with time, since the particles associate and agglomerate, changing the number of particles *n* which determines translational kinetic energy. As in our previous publications,^24^,^25^ we used QCMD since we noted that our application of the classical molecular dynamics was not effective in producing ordered BN structures, resulting in mainly amorphous nanostructures. Our analysis will focus on elucidating the optimal conditions and precursors for the volume plasma synthesis of BNNTs as well as h-BN flakes and cages in high yield/high quality, *i.e.* with the least number of defects.

## 2. Computational methodology

Consideration of the electronic structure is essential to perform QCMD studies of the nanostructure self-assembly mechanisms in plasma environments when electron delocalization plays a role,^29^ as is the case for any h-BN structure. First-principles density functional theory (DFT) is computationally too expensive for its direct use in QCMD, and therefore we employed a more economical density-functional tight-binding (DFTB)^30^ approximation instead. Such QCMD, based on the DFTB method to describe electronic structure, led to a series of BNNS self-assembly simulations^24^–^26^ that were in qualitative agreement with experimental observations. DFTB is an approximate DFT method and utilizes an optimized valence-only minimal linear combination of atomic orbital (LCAO) basis set in combination with a two-centre approximation for Hamiltonian and overlap matrix elements.^30^ In addition, it utilizes a diatomic repulsive potential that describes core–core repulsion and corrects for double-counting of electron–electron coulombic and exchange interactions. It is roughly two to three orders of magnitude faster than traditional DFT, while often being competitive in terms of molecular geometries, energetics, and vibrational spectra. In the present work, we use the “matsci-0-3” parameter set^31^ for the electronic and diatomic repulsive potentials between chemical elements B, N, and H. Orbital occupation numbers are determined for each time step using a Fermi–Dirac distribution with finite electronic temperature *T*,^32^ chosen to be equal to the targeted nuclear temperature which is controlled by a Langevin thermostat.

Molecular dynamics simulations were performed by numerically integrating Newton's equations of motion on the instantaneously computed adiabatic DFTB potential energy surface, using a time interval of 1 fs in a velocity Verlet integration scheme. The Langevin thermostat was applied every 10 fs with a friction coefficient of 0.01 fs^–1^ thus keeping the total kinetic energy of the system, and therefore its temperature *T*, at an approximately constant level *E*_k_ = 3/2*n*_i_*k*_B_*T*. The fluctuations in kinetic energy at *T* = 2000 K were in the range of ±0.01 eV per atom, a reasonable performance for a Langevin thermostat. The thermostat is applied to all atoms in the system, whether they aggregated into clusters, in which case their kinetic energy contains vibrational, translational and rotational components, or remained in atomic form, in which case their kinetic energy is only translational. The temperature adjustment results in an approximately constant temperature *T*, and also increases the vibrational and rotational motions of the clustered and agglomerated particles with increasing choice of the desirable “target” instantaneous temperature. As the association, clustering and agglomeration of atoms and nanoparticles proceed with time, the pressure, which is merely defined by the translational energy of the emerging particles, is significantly decreasing due to both reduced effective number of particles and their total translational energy, and is quite difficult to calculate and maintain in the given reactive environment. Further discussion on the role of thermostats during self-assembly processes can be found in ref. 33. QCMD simulations were run at temperatures typically encountered in certain areas of plasma volume, namely 1500, 2000, 2500, 4000, and 6000 K, for a total duration of 1 ns in each case. At the beginning of each trajectory, particles were given random velocities which correspond approximately to the Maxwell distribution at the desired temperature *T*, followed by application of the thermostat with the same temperature. Periodic boundary conditions in all three dimensions were applied to the cubic simulation cell with typically 11 nm lateral dimension, containing about 1300 atoms. The *Γ*-point approximation for *k*-space sampling was employed throughout the simulations. The coulombic interactions between periodic images were evaluated using the standard Ewald summation method. Before agglomeration happens in the course of the trajectory, using particle density of *n*_i_ = 1.0 × 10^21^ cm^–3^, typical for atomic precursors in our simulations, the kinetic energy supplied by the thermostat yields an initial pressure of *p*_i_ = *n*_i_*kT* ∼ 1.38 × 10^4^*T* Pa, where *T* is in units of K. Thus, at 2000 K the initial pressure reaches about 270 bars. The large initial number of particles in the box, resulting in high pressure, is chosen for the sake of promoting reactive collisions, accelerating the self-assembly processes during a short simulation time of 1 ns.

Since the computational effort in conventional DFTB scales cubically with the number of pseudo-atomic orbital basis functions, with the largest simulated systems requiring close to 10 000 basis functions, and because one million gradient evaluations are required during 1 ns simulation time, we decided to utilize the divide and conquer (DC) technique^34^ in our QCMD simulations, as adapted for DFTB by Nakai *et al.*,^35^ who named the resulting method DC-DFTB. The DC method was first developed by Yang and Lee in 1995 (ref. 34) and has been widely employed to achieve linear scaling of quantum chemical methods including DFT,^36^ Hartree–Fock,^37^ Møller–Plesset Perturbation Theory,^38^ and Coupled-Cluster Theory.^39^,^40^ The DC technique allows linear-scaling of DFTB theory to treat large systems with many basis functions by dividing the three-dimensional space into non-overlapping subsystems, called “central regions”, and overlapping “buffer regions” that include the associated central regions.^34^ In each of these regions, the molecular orbitals (MOs) are constructed from the pseudo-atomic orbitals using the standard LCAO approach by solving the Kohn–Sham equations for this part of space only. In the present simulations, the central regions are cubes with side lengths of 5 Å and the buffer regions are constructed as spheres around atoms in the central regions with a buffer radius of 5 Å. These choices are the default settings in the DC-DFTB-K code.^35^ The density matrix of the total system is then constructed as a partitioned sum of subsystem contributions by applying a common Fermi level in the Fermi–Dirac distribution function^32^ that is determined from the total number of electrons. This density matrix is subsequently employed in the calculation of the total energy of the entire system, and of the analytical energy gradients using the usual Pulay energy-weighted density matrix term as in standard DFTB.^35^ The real-space partitioning scheme of DC lends itself to efficient parallelization, and we performed each trajectory reported in this work on 16 CPU cores, utilizing the MPI protocol as implemented in the DC-DFTB-K code. Even when using this accelerated version of DFTB, one set of input parameters and configurations required a total of thousands of CPU hours (several days on 16 CPU cores) to reach 1 ns of the system evolution on Intel Xeon CPUs with 3.2 GHz clock speed.

## 3. Overall simulation results

### 3.1 Relationship between reactants and products

The simulation of the BNNS synthesis under plasma conditions requires a choice of the precursors that might be the atomic and/or molecular species present in the plasma torch^21^ or arc discharge^28^ experiments. We selected 11 configurations of B, N, and H containing precursors listed in Table 1, at the aforementioned five different target temperatures in the range between 1500 and 6000 K. The precursor systems can be divided into two groups, namely one group which does not contain hydrogen (group A), and another group where hydrogen is included (group B). Each of these precursor groups can be divided into two subgroups, those containing initially only atomic precursor species (A1, B1), and those which initially already contain chemical compounds (A2, B2).

**Table 1:** Various precursors, their contents and corresponding symbols used in the text. Groups and subgroups are explained in the text. The content weights are normalized to unity, and each precursor group contains ∼1300 atoms in total

Group	Subgroup	Content	Symbol
A	A1	1/2B 1/2N	B–N
		1/3B 2/3N	B–2N
		2/3B 1/3N	2B–N
		1/4B 3/4N	B–3N

	A2	BN	BN

B	B1	1/3B 1/3N 1/3H	BNH/111
		1/4B 1/2N 1/4H	BNH/121
		1/2B 1/4N 1/4H	BNH/211

	B2	4/5BN 1/5H	BN–H
		HBNH	HBNH
		B_3_N_3_H_6_	Boraz.

**Fig. 1:**
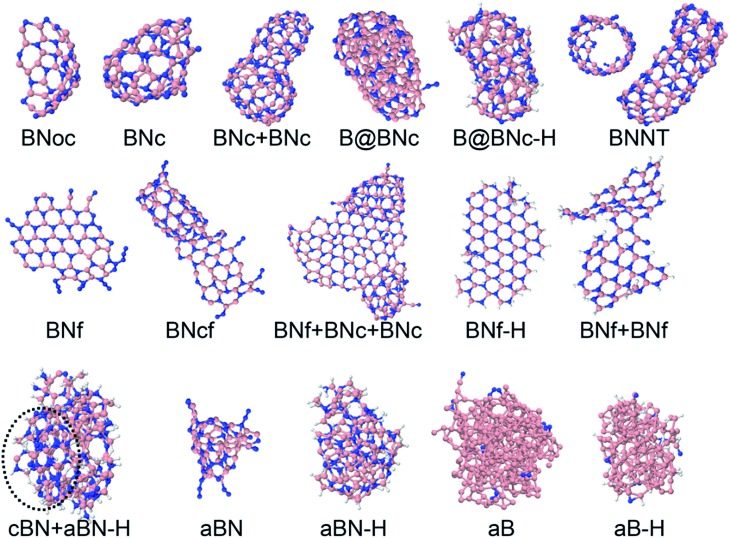
Representative self-assembled products and their associated product labels at the final snapshot of actual QCMD trajectories. Turbostratic BN (t-BN) and tetrahedral-amorphous BN (ta-BN) structures are included in aBN.

The precursors in Table 1 self-assemble, after 1 ns of simulations at a given temperature, into a variety of nanostructures of various sizes, as shown in Fig. 1, which displays representative clusters selected from the final snapshot of actual trajectories. We are most concerned about BNNTs, BN cages (BNc), BN flakes (BNf), cubic BN (cBN), and amorphous BN (aBN). Occasionally we find boron clusters encapsulated inside other BNNSs, which we indicate by the symbol “B@”. At high temperatures of 4000 and 6000 K we find predominantly amorphous boron clusters (aB). The complete glossary for the labels used in Fig. 1 can be found in Appendix A.

Using the symbols given in Table 1 for the content of various precursor configurations, as well as labels for the obtained self-assembled products given in Fig. 1, we can construct precursor–product maps for the various BNNSs that were obtained from various precursors. Fig. S1 of the ESI† shows such a map for the temperatures of 1500 and 2000 K.

In our simulations, we find that structures based on h-BN far outweigh structures that contain c-BN, and are most notably made up of semi-ordered structures (cages, flakes, nanotubes) rather than amorphous clusters. We apply the term semi-ordered since the self-assembled BNNSs contain defects such as pentagons, heptagons, and other rings, as well as non-alternating B–B and N–N bonds, however, to a relatively small degree. Ohta *et al.*^26^ and we^24^,^25^ have previously noted that alternating the order of B–N bonds naturally emerges in QCMD simulations, following thermodynamic stability trends: B–N bonds between sp^2^-hybridized B and N atoms are stronger than N–N or B–B bonds, and the nascent BNNSs undergo annealing, rearranging energetically unfavourable bonding situations into more favourable ones. Since the main goal of the present study is to identify the favourable conditions in the high-pressure plasma concerning temperature and atomic content of precursors that possibly lead to a “quality” synthesis of the BNNSs in plasma, it becomes a necessity to define means of quantification for their structural “order”. The justification for and definition of such a “parameter of the synthesis quality” (PSQ) will be introduced in the following paragraph.

### 3.2 Relationship between reactants and synthesis quality

Hexagons comprised of all-alternating B–N bonds are the common structural subunit for both h-BN- and c-BN-based BNNSs. It is therefore natural to evaluate their numbers *via* a ring counting algorithm which we adopted from the TINKER modelling package.^41^ For a given configuration, at any time during the evolution of plasma precursor species to BNNS clusters and aggregates, the maximum number of hexagons that can be formed from either trivalent (sp^2^) or tetravalent (sp^3^) hybridized B and N atoms present at a given simulation time *t* is1
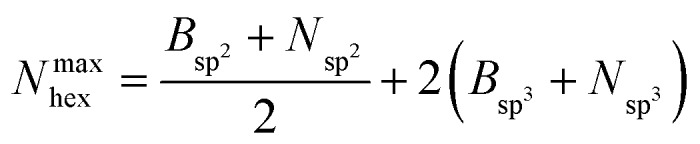
where all quantities in eqn (1) are functions of *t*. In the atom count of eqn (1) we consider only those B or N atoms that are bound to their “complementary” element partners (B to N, or N to B), excluding all atoms with homo-elemental B–B or N–N bonds, as is the case in non-defective BNNSs. We call these atomic species “ideal” hybrids. In addition, in order to emphasize the self-assembly, we exclude precursor species and count only those ideal hybrids contained in clusters with more than 10 atoms. If *N*_hex_ is the actual number of hexagons at time *t*, which have equal number of N and B atoms, we loosely define a measure for the probability of hexagon formation at *t* as:2
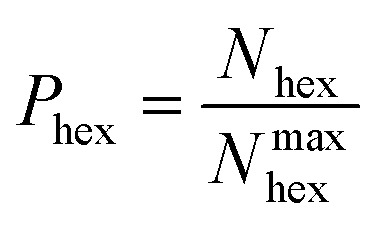
We note here that, because the factor multiplying sp^3^-type atoms in eqn (1) is 4 times larger than the factor for sp^2^-type atoms, the number of sp^3^ rings might significantly influence the hexagon formation probability *P*_hex_ as defined in eqn (2), even if the number of sp^3^ atoms is small in comparison to the sp^2^ ones. We will later return to this somewhat problematic issue.

For precursor species that contain hydrogen atoms, we often find that 2D planar, BNf structures in Fig. 1 are terminated by hydrogen atoms at their edges, preventing them from forming closed nanostructures. This situation requires accounting for sp^2^ atoms with a single bond to a hydrogen atom in addition to the other two complementary BN bond partners (two Ns for a B atom, and two Bs for a N atom), in order to accurately count the number of edge sp^2^-type atoms. In the case of sp^2^ edge atoms without hydrogen, besides their bonding to the two complementary atoms, these are also identified by requirement that their bond angle is in the range 120° ± 30°.

Finally, in the case of the borazine precursor subgroup, an additional counting restriction was applied. Namely, a borazine molecule already contributes initially (at *t* = 0) one ring to the hexagon count, affecting *P*_hex_. Hence, for the borazine precursor subgroup, we only count the hexagon number of clusters containing more than 12 atoms (rather than 10, as with other precursor types), which automatically excludes the hexagons associated with unreacted precursor borazine.

**Fig. 2:**
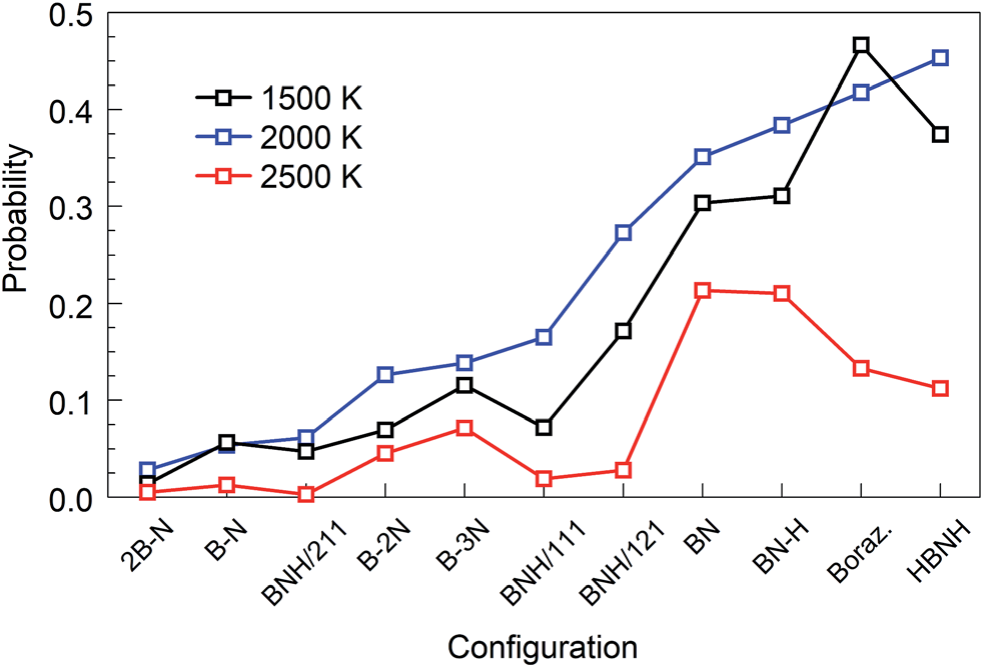
Parameters of the synthesis quality (PSQs) for all initial configurations and selected temperatures at *t* = 1 ns. Precursor subgroup species are ordered along the abscissa following the increasing order of PSQ at 2000 K.

**Fig. 3:**
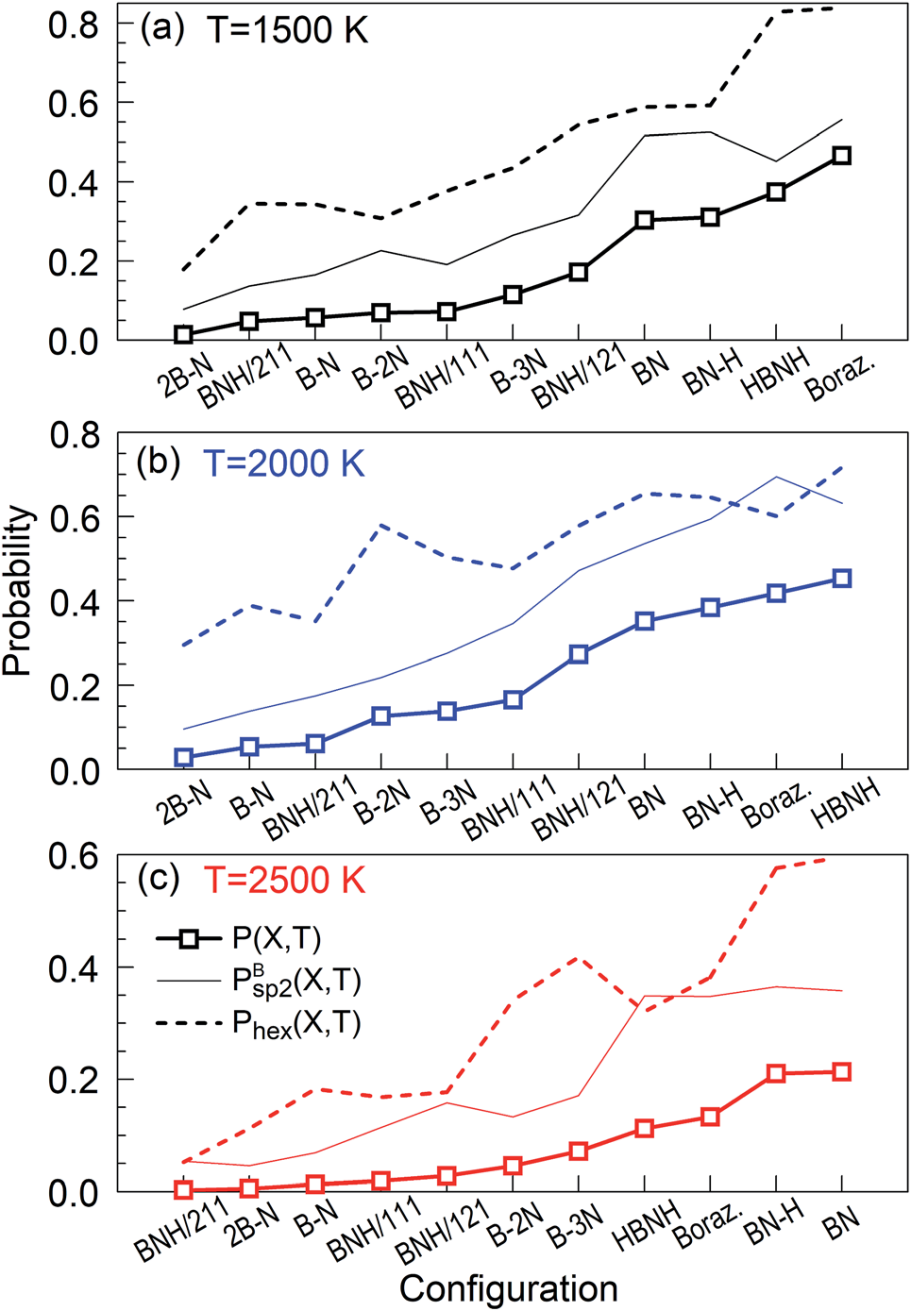
PSQs, ordered to produce monotonic curves for each temperature at *t* = 1 ns. The factors of the PSQs are the hexagon probabilities and *B*_sp^2^_ probabilities, explained in the text. Each tick at the horizontal axes is associated with one precursor subgroup with the symbol indicated below it.

However, as mentioned before, the hexagon formation probability defined in eqn (2) does not discriminate between h-BN and c-BN species. In the context of the technologically more relevant BNNT, BNf, and BNc structures, we are more interested in the formation of h-BN-type BNNSs. Thus, in order to convert *P*_hex_ to a probability for the creation of an h-BN structure, we multiply *P*_hex_ by the probability for the formation of a boron sp^2^ ideal hybrid, 
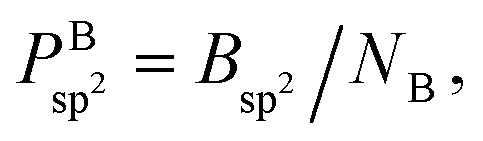
 where *N*_B_ is the total number of boron atoms in the simulation box. Thus, we estimate the probability of synthesis of a h-BN structure from a particular precursor subgroup *X* and at temperature *T* by3
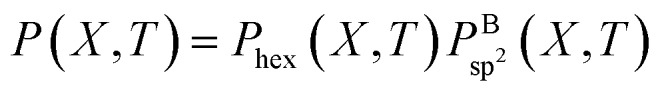
we refer to *P*(*X*,*T*) as “parameter of the synthesis quality” (PSQ), and the values of *P*(*X*,*T*) for various temperatures *T* = 1500, 2000 and 2500 K and all initial precursor configurations are shown in Fig. 2. The order of the various *X* precursor subgroups at the abscissa is chosen so that *P*(*X*,*T*) is a monotonically increasing function of *X* for *T* = 2000 K. *P*_hex_(*X*,*T*) and 
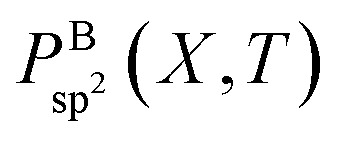
 are shown together with their PSQ product *P*(*X*,*T*) in Fig. 3. The orders of the *X* precursor subgroups along the abscissa are adapted for each temperature so that *P*(*X*,*T*) is a monotonic function of *X*. Obviously, for these adapted *X* orders, neither of the two component factors is monotonic of *X*, these rather act in opposition to result in a monotonic dependence.

The “winners” among all tested temperatures are precursor subgroups that contain the BN functional group, *i.e.* BN, BN–H, borazine and HBNH. However, their mutual “success order” depends on the temperature. Thus, the “winner” at 1500 K is borazine, at 2000 K it is HBNH and at 2500 K the “winner” is radical BN, nearly tied with BN–H. The worst positions in ranks defined by *P*(*X*,*T*) are the atomic precursors with high content of boron, such as 2B–N and BNH/211. The B–N precursor subgroup is also ranked low. However, increased content of N, like in BNH/121, B–2N and B–3N, places these configurations in the middle rank positions in Fig. 2 and 3. It is also clear that there is not a large difference in synthesis efficiency of the h-BN nanostructures between 1500 and 2000 K, though efficiency and quality at 2000 K is for almost all precursor configurations higher. However, with increasing temperature, the synthesis efficiency decreases for all configurations. For instance, at 2500 K, the efficiency for the HBNH subgroup drops to one third of its efficiency at 2000 K. With further increase of temperature, the efficiency of the h-BN nanostructure synthesis further decreases, being almost negligible at 4000 and 6000 K, where boron clusters (aB) dominate the products, surrounded by an atmosphere of N_2_. Considering all our simulation results, we attest the temperature of 2000 K the status of a “winning” temperature.

## 4. Discussion of the results

We now analyse the simulations in detail and discuss the meaning of our findings. We will focus on the “winning” precursor configurations in Fig. 2 (BN, BN–H, borazine and HBNH), in particular for the “winning” temperature of 2000 K, although the other cases will also be briefly described.

### 4.1 BN precursor

The cubic simulation box with a lateral dimension of 11.34 nm was filled with 729 regularly distributed BN molecules in random orientations. The molecules are initially sufficiently separated such that the total energy is equal to the sum of all precursor potential energies.

**Fig. 4:**
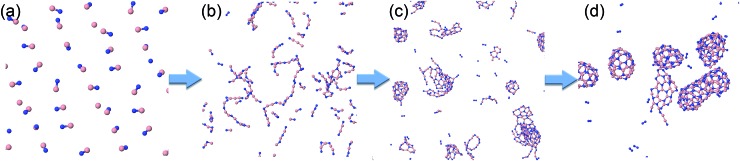
Typical structural evolution of the BN precursor system at 2000 K during the simulation time of 1 ns: (a) 0 ps, (b) 30 ps, (c) 130 ps, and (d) 1 ns.

Typical phases of the structural evolution of the system at *T* = 2000 K during 1 ns are shown in Fig. 4 and Movie S1 in the ESI.† In the first phase, the BN molecules form BN chains, not necessarily in an all-alternant manner since one BN molecule can react with another BN to form either a new B–N, B–B, or N–N bond. Aside from the difference in elemental composition, the chain formation is highly reminiscent of the situation of carbon plasma.^29^ Also, similar to the case of carbon, the chains begin to branch, creating Y-junction structures with central sp^2^-hybridized centers, and then start joining into various 2D and 3D structures. We note that the “pentagon-first” rule, theoretically observed and described for carbon nanostructure self-assemblies,^42^ does not seem to hold for the BNNS self-assembly, where alternation of B–N atoms and thus even number of atoms is preferable. These more complex clusters further reorganize internally as well as undergo coalescence to yield various well-organized nanostructures, such as BNc, BNf, and BNNT, following similar ring-condensation mechanisms to the case of carbon fullerene formation.^29^

**Fig. 5:**
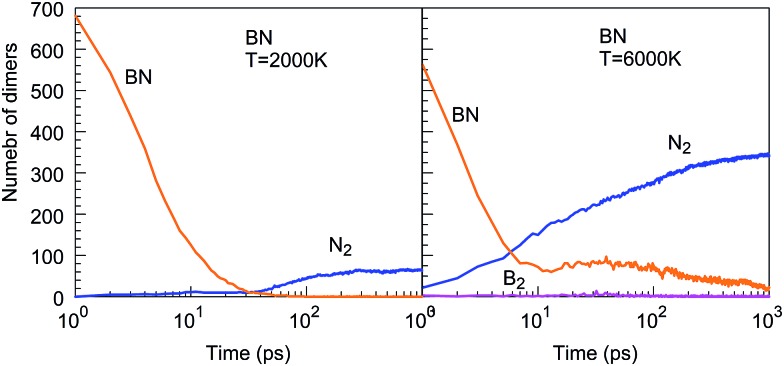
Evolution of diatomic molecular species from initial BN precursor molecules, at 2000 and 6000 K.

As is also apparent from Fig. 4, during the time evolution, N_2_ molecules appear in the trajectory, although they were not present in the initial precursor mix. We attribute this general tendency to the extremely high bond dissociation energy of the N–N triple bond in the dinitrogen molecule. The evolution of BN and N_2_ molecules as a function of time is shown in Fig. 5 for the “winning” temperature of 2000 K and for the highest temperature studied, namely 6000 K. The evolution of diatomics at 1500 and 2500 K is qualitatively similar to the one at 2000 K, while their evolution at 4000 K resembles the one at 6000 K. Thus, for 2000 K, all BN molecules disappear from the species mixture, as they typically become part of the organized BNNSs. Our analysis shows that about 20% of BN molecules dissociate, which leads to about 10% of N_2_ (in comparison to the initial BN number). At a temperature of 6000 K, the process of gathering BN diatomics into chains and BNNSs is significantly suppressed, which leads to a residual of only about 5% free BN diatomics after 1 ns. However, the majority of BN precursors dissociate at this high temperature, leading to an increased number of associated N_2_ molecules, as was postulated from experimental results.^23^ A small number of “free” boron atoms associate into B_2_, and the rest form large boron clusters, which contain only a very small amount of nitrogen, typically on the cluster surface.

**Fig. 6:**
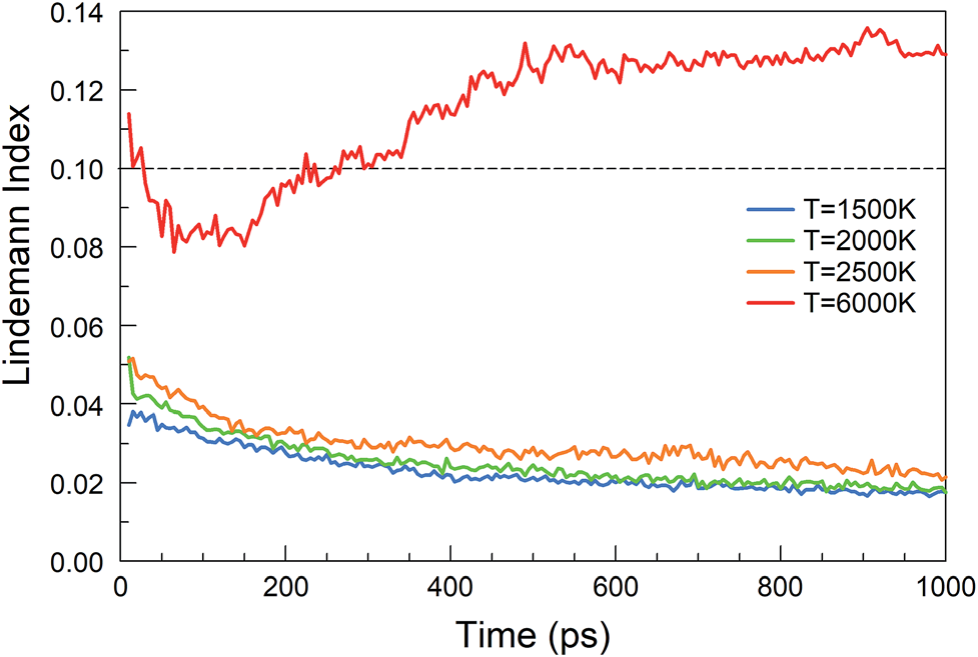
Lindemann indices as a function of time during the evolution of the BN precursor system for various temperatures.

The Lindemann index^43^ is a useful parameter to identify phase changes, and it is shown in Fig. 6 for various temperatures of the BN simulations. Usually an index greater than 0.1 is associated with the liquid phase, whereas a value smaller than 0.1 signifies the presence of a solid phase.^44^ At temperatures between 1500 and 2500 K, boron and nitrogen self-assemble into solid structures. However, at 6000 K, almost pure boron clusters are formed in an atmosphere of gaseous N_2_ molecules. The Lindemann index is always above 0.1 after 300 ps, consistent with the observed molten boron nanoclusters. We note that the melting temperature for bulk boron is 2350 K, whereas the melting temperature of bulk h-BN is 3246 K, while nanometer-sized particles have generally lower melting temperatures due to the Gibbs–Thomson effect.^45^

**Fig. 7:**
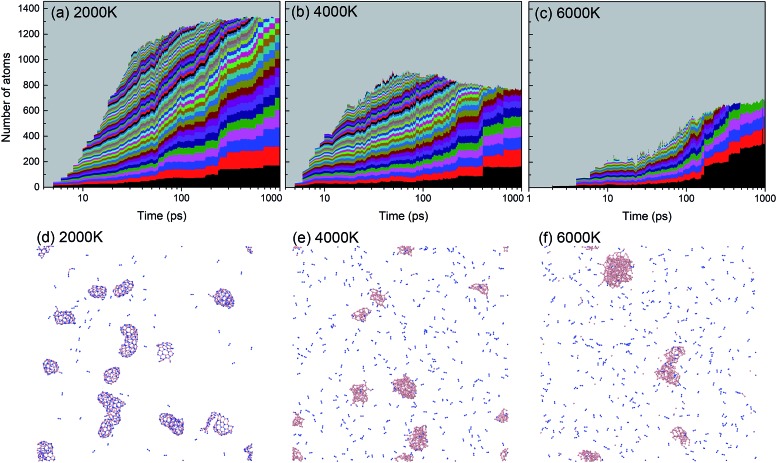
Histogram of cluster sizes based on clusters containing more than 10 atoms of the BN system at (a) 2000 K, (b) 4000 K, and (c) 6000 K. Structures created after 1 ns are respectively shown in (d–f). At 2000 K mainly ordered h-BN-based BNNSs are created, while at 4000 and 6000 K mainly boron clusters (aB) appear, surrounded by an atmosphere of N_2_ molecules.

A histogram of cluster sizes based on clusters containing more than 10 atoms, for the trajectories at 2000, 4000 and 6000 K, is presented in Fig. 7. The latter two histograms for the higher temperatures show mostly boron clusters. The height of each box frame in the figure represents the total number of atoms in the system, *i.e.* the sum of atoms in clusters, various small molecules and atoms. Each color belt represents a cluster containing 10 or more atoms, and the height of a belt measures the number of atoms in the respective cluster. We note that a given color does not necessarily indicate the evolution of the same cluster. For instance, if two clusters coalesce into one larger cluster, the new cluster will be assigned the color of the larger of the previous two. All aggregates containing less than 10 atoms are collectively included in the grey area. The appearance of belts after several picoseconds indicates the aggregation of BN molecules to form larger structures. The qualitative behavior of cluster growth shows two phases. In the first phase, small molecules are rapidly consumed until a maximum number of individual clusters is reached. This phase is associated with a positive curvature in the cluster size envelope, and lasts until ∼40 ps for 2000 K, and only until ∼10 ps for 4000 and 6000 K. Then, in the second stage, these clusters coalesce to form clusters of larger sizes, and at the same time, the number of clusters decreases, following an Ostwald ripening process. The curvature of the cluster size envelope is now positive, and the number of atoms in the small molecules (grey part) is almost constant in this stage or increases again at 4000 K. In all cases, we do not observe the growth of a single largest cluster, as was observed in the case of benzene combustion, where a single carbon cluster could consume all other carbon clusters present in the simulation box.^46^

A significant effect of temperature on cluster sizes is apparent from the comparison of Fig. 7a–c. At 2000 K, ∼10 clusters of similar size containing ∼80 to 200 atoms survive until the end of the simulation at 1 ns, and together they consume about 90% of all precursor atoms. As the temperature increases, the number of initially created clusters as well as the number of surviving major clusters decreases, the average size of the clusters increases, and the total number of atoms consumed in the major clusters decreases. At 4000 and 6000 K, these clusters contain mainly boron atoms. The larger readiness of liquid boron clusters (“droplets”) to coalesce at higher temperatures leads to a higher aggregation probability of clusters, which explains the larger sizes of the final boron clusters. The increased conversion of nitrogen atoms to N_2_, not included in the cluster size count, with increased temperature explains the drop of consumed atoms to roughly 50% at 6000 K (pure boron clusters survive). The evolution trajectory at *T* = 4000 K can be found in Movie S2 in the ESI.†

**Fig. 8:**
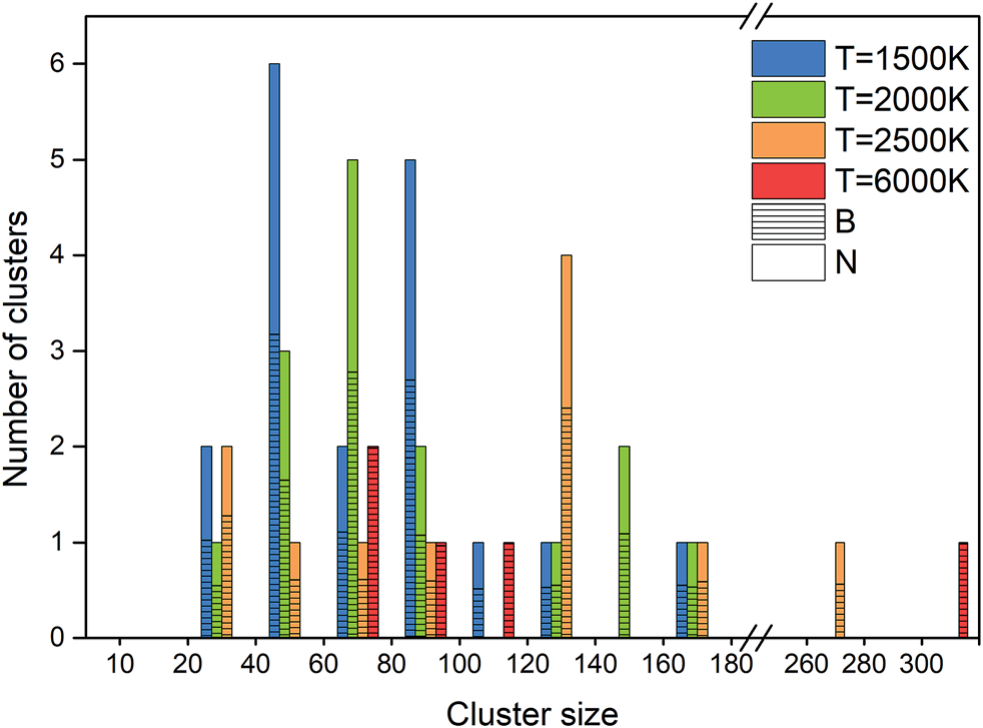
Final atomic content, size and number of clusters at 1 ns, at 2000 K.

An alternative way of presenting the cluster sizes at the end of the simulation after 1 ns is presented in Fig. 8 as a bar graph with bins, *i.e.* intervals of cluster sizes, for four temperatures. The atomic content of each cluster is of particular interest, since an ideally organized BNNS contains 50% B and N atoms each. Therefore, we also show the B and N contents, averaged over the clusters contained in each size interval. The boron atomic content in the total cluster size is indicated by bars shaded with horizontal lines, the rest being nitrogen. The number of clusters of a particular size interval (in a bin) is indicated at the ordinate.

As discussed earlier and shown in Fig. 7, lower temperature generally leads to smaller surviving clusters at the end of the simulation time, due to lower mobility and lower cluster reactivity. Fig. 8 naturally confirms this trend, with the cluster size distribution shifted toward larger sizes with increasing temperature. The number of clusters at 2500 K is smaller for almost all sizes. The content of B and N in these clusters is approximately equal, with a slight dominance of boron. Consistent with the previous analysis, for 6000 K, a relatively small number of larger, pure boron clusters can be seen. This is illustrated in Fig. 7d–f, showing the qualitative difference of the surviving clusters for three temperatures.

**Fig. 9:**
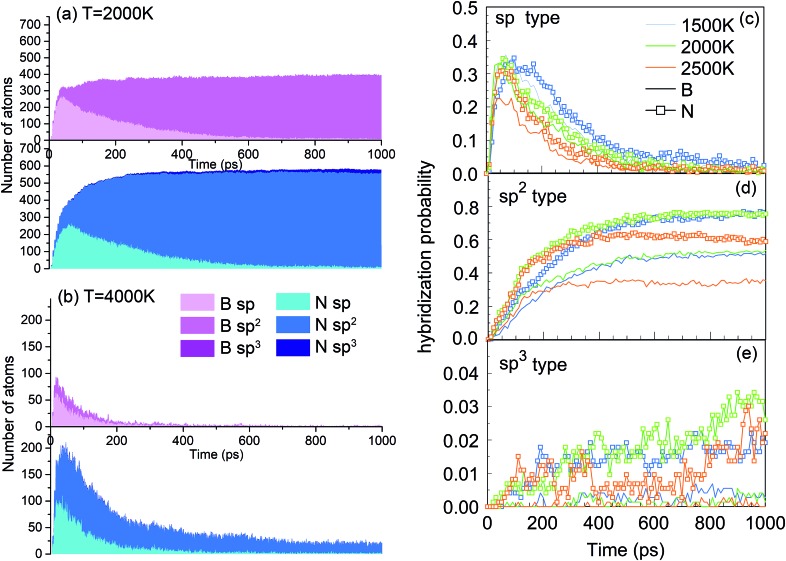
Number of ideally hybridized sp, sp^2^ and sp^3^ atoms for B (pink) and N (blue) atoms at (a) 2000 K and (b) 4000 K, as a function of time for the BN precursor system. Probabilities of formation of (c) sp, (d) sp^2^ and (e) sp^3^ hybridized B and N atoms at various temperatures, as a function of time.

One of the synthesis quality assessment factors, used in Section 3, is the hybridization of both B and N atoms in the system. As discussed previously in Section 3, we will deal only with the “ideal” hybrids, B or N atoms bonded only to their elemental counterpart. In the case of B, “ideal” sp, sp^2^ and sp^3^ hybrids mean two, three and four N atoms bonded to a B atom, respectively, and *vice versa* for N. Thus, the ideal sp hybridized atoms characterize fully element-alternating BN chains, the sp^2^ atoms are a signature of h-BN formation, and the sp^3^ atoms signal c-BN-type structures. Their populations as a function of time are shown in Fig. 9a and b. Obvious is the creation of chains, *i.e.* sp structures, in the first picosecond, with their population decreasing after a few tens of picoseconds, due to the process of more complex nanostructure creation. While the sp^3^ hybrid population increases with time, they never contribute a few percent. The wide bands in Fig. 9a show that, at 2000 K, the sp^2^ hybrids are by far dominant, in comparison to other hybrids, whose number is negligible after a few hundreds of picoseconds. Still, the number of nitrogen sp^2^ hybrids is about 50% larger than the number of boron sp^2^ hybrids, characterizing the existence of numerous defects in the structures. Thus, the smaller number among these, which describes the number of sp^2^ hybridized B atoms, indicates the size of defectless h-BN-type BNNSs. This is the reason to use the probability of the sp^2^ boron creation for constructing the PSQ parameter in Section 3, as previously explained. The evolution of BN precursors at 4000 K (represented in Fig. 9b) results in predominantly boron clusters. It shows that the system passes through a phase of a small number of sp and sp^2^ “ideal” hybrids for boron and nitrogen, but rapidly declines after a few hundreds of ps, until almost no N-bonded B atoms exist, confirming our visual qualitative observation that the system at 4000 K agglomerates mainly boron in boron clusters, while nitrogen mainly associates into N_2_ molecules. The probabilities of forming B and N atoms of different hybridizations *x* (*x* = sp, sp^2^, sp^3^) are calculated as *n*_Bx_/*n*_B_ and *n*_Nx_/*n*_N_, respectively. *n*_Bx_ and *n*_Nx_ are the actual atom numbers of “ideal” hybridization type *x* for B and N, counted in the system, while *n*_B_ and *n*_N_ are the total number of B and N atoms in the system, respectively. For instance, for the BN precursor system, *n*_B_ = *n*_N_. The evolution of the probabilities at different temperatures is shown in Fig. 9c–e, where one can see a direct correlation with Fig. 4: The fast creation of BN chains is depicted by a sharp increase in the number of sp-hybridized atoms at the beginning until ∼40 ps (Fig. 9c). The phases that follow are chain branching and creation of h-BN structures as represented by the gradual increase in the number of sp^2^ hybridized atoms accompanied by a decrease in the number of sp atoms (Fig. 9c and d).

**Fig. 10:**
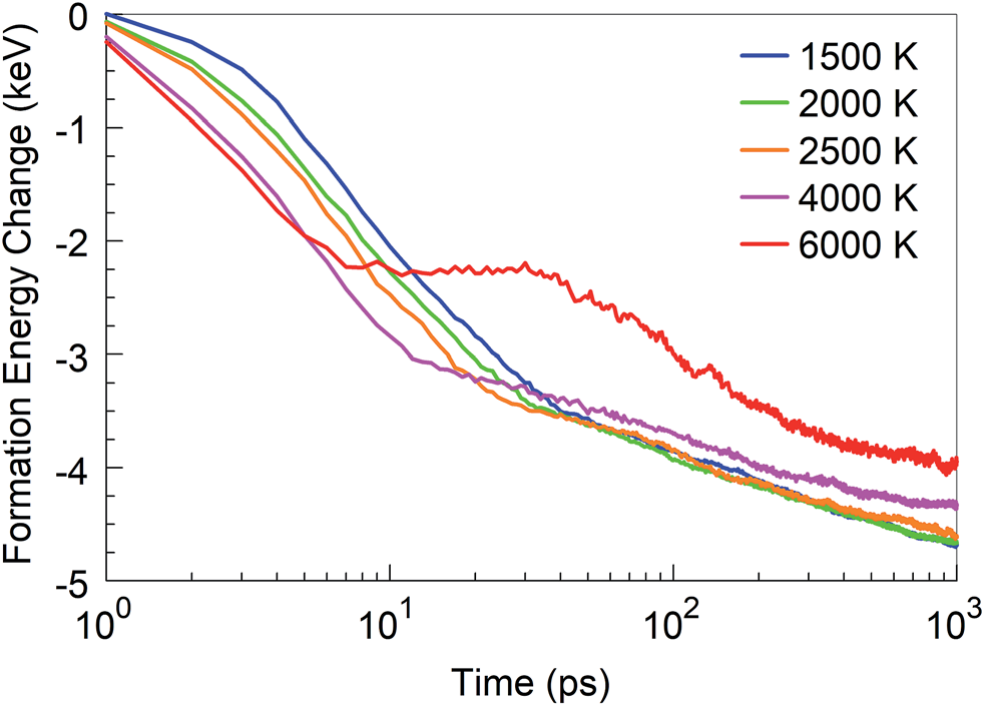
Formation energy change of the system at time *t* from *t* = 0, at various temperatures.

In addition to the structural analysis, it is interesting to analyse the energetics of the self-assembly processes at the various temperatures. For this purpose, we computed the formation energy of the system as a function of time, as it indicates the energy associated with the self-assembly processes, using the total potential energy of the system at *t* = 0 as the reference. The formation energies for four selected temperatures are shown in Fig. 10. As one might expect, it is immediately obvious that the energy of formation is monotonously decreasing with time, indicating the exothermicity of the self-assembly process. At temperatures in the 1500–2500 K range, three different slopes are visible on the log-linear scale: the first one lasts for a few picoseconds, the second one for a few tens of picoseconds, and the third extends to the end of the simulation. These three slopes, or domains, characterize, for the 1500 to 2500 K range, the principal phases of the system evolution: (1) during the first few picoseconds, BN chain formation and dissociation of BN molecules occurs; (2) in the following few tens of picoseconds, short chains form longer chains with *Y*-junctions, branch further, and create 2D structures and cages, and react with residual nitrogen atoms in floating N_2_ molecules; and (3) structural rearrangements of the created structures, defect healing, Ostwald ripening, flake folding into the tubes, and other large-scale, slow transformation processes occur. We note that the energy profile for 6000 K deviates qualitatively from this behaviour. Here, in the first 10 ps, BN chains are formed, similar to the case of lower temperatures. However, in the second phase, a segregation process follows during which predominantly N_2_ molecule and boron cluster formation occurs. This process is energy-neutral, and followed by a phase during which smaller boron clusters aggregate to larger boron droplets. The formation energy for 4000 K largely follows the trend of the curves at low temperatures, except that the first phase here proceeds much faster. The formation energy at 1 ns is smaller at 4000 K than that at lower temperatures due to the formed boron clusters and N_2_ molecules rather than BN structures.

### 4.2 BN–H precursor

BN–H is the simplest of all studied precursor subgroups in Table 1 that include hydrogen. It differs from the BN precursor by the addition of a number of hydrogen atoms to the mix of BN molecules. In particular, we “mixed” 580 BN molecules and 148 hydrogen atoms in the cubic simulation box with a dimension of ∼11 nm, ensuring no interaction between the precursor species. The time evolution of this system and the one with BN precursors turns out to be surprisingly similar. The final products are also similar, except that the nanostructures built from the BN–H precursor system always contain some amount of H at their external surface, as indicated in the precursor–product map of Fig. S1.† The transformations preceding clustering undergo similar phases as displayed in Fig. 4, including the formation of chains, to the final BNc-, BNf-, and BNNT-like products, all with some hydrogens, typically saturating dangling bonds at the open edges of the BNNSs. The dimer content, as shown in Fig. S2,† is also quite similar to the case of the BN precursor (Fig. 5), except that there is a number of BH dimers in the BN–H case at 6000 K. The observed role of hydrogen in the BNNS self-assembly process is similar to the previously reported simulations of the effect of hydrogen contents on carbon nanostructure formation in benzene combustion.^47^

The size distribution and final atomic content of the clusters for various temperatures are shown in Fig. S3.† A relatively small amount of H (up to 10% on average) is present in all clusters. Interestingly, the largest clusters are obtained at 2000 K, with sizes greater than 300 atoms. At this temperature, the content of B dominates slightly over N + H. Generally speaking, the boron content increases with temperature, and absolutely prevails at high temperatures of 4000 and 6000 K. However, hydrogen at the external surface of the boron clusters slows down the agglomeration of smaller to the larger boron clusters, which explains the smaller aB–H clusters at 6000 K when compared to the aB clusters produced by the BN precursors at the same temperature.

The hybrid content in Fig. S4† is very similar to the one in Fig. 9 for the BN precursors. The energy diagram also looks similar to the one in Fig. 10, including the slopes of the curves. The creation of cages, flakes and BNNT for BN–H also shows similar trends to the BN case. These similarities explain the very similar PQS values for BN and BN–H precursor systems in Fig. 2 and 3.

### 4.3 Borazine (B_3_N_3_H_6_) precursor

**Fig. 11:**
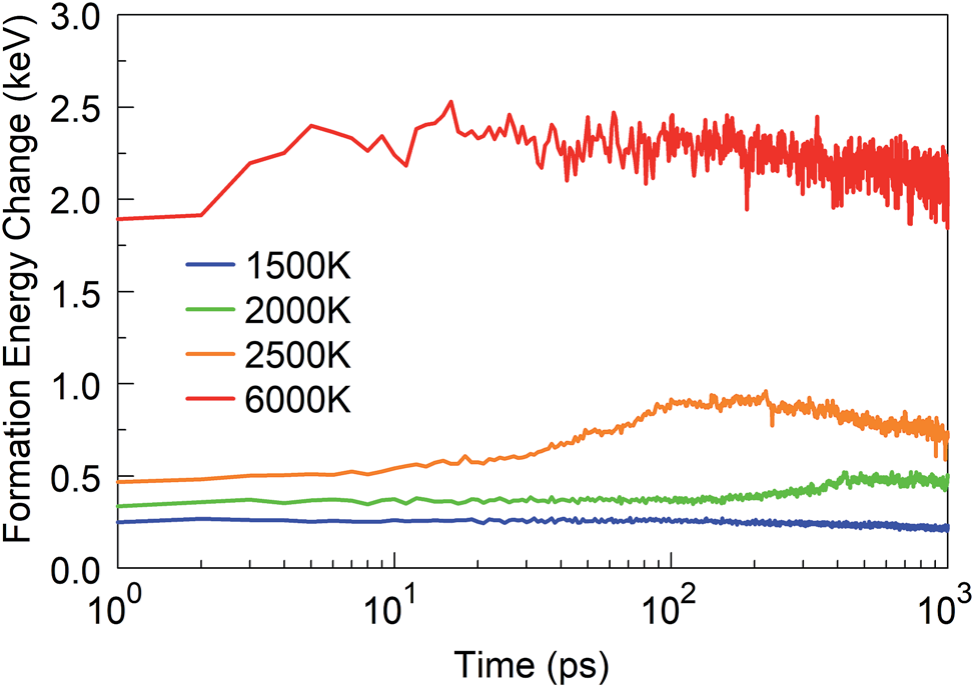
Formation energy change of the borazine system at time *t* from *t* = 0 at various temperatures.

We started from 125 borazine molecules, which were placed in the cubic simulation box with a dimension of ∼11 nm in random orientations, ensuring no interaction between the precursor species. Clearly, borazine is a thermodynamically stable molecular precursor which can be expected to react more slowly than the previous radical-containing precursor groups BN (the BN molecule has a triplet ground state) and BN–H. We find that the evolution of the formation energy, as shown in Fig. 11, correspondingly differs substantially from the previous three-phase behavior, and does not show overall exothermicity of the self-assembly processes.

**Fig. 12:**
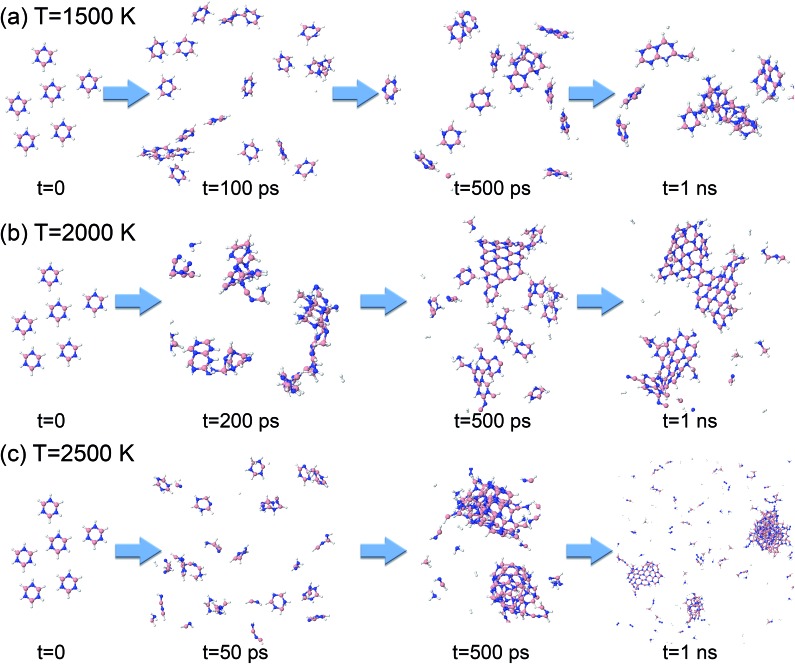
Phases of evolution of the borazine precursor system at (a) 1500 K, (b) 2000 K, and (c) 2500 K.

Starting from 1 ps, the formation energy change at 1500 K is flat until about 100 ps. Indeed, from Fig. 12a we can see that at 100 ps, no chemistry has occurred and the molecules simply have undergone diffusion. At 500 ps the formation energy increases (in absolute value) due to aggregation of some borazine molecules into flat, condensed-ring structures. Such association processes continue, and at 1 ns, some amorphous BN structures of turbostratic nature (t-BN) are formed besides BNf–H, which explains the further decrease of the formation energy.

At a higher temperature of 2000 K (Fig. 12b and Movie S3 in the ESI†), the association of borazine molecules into flat structures starts earlier, namely from about 200 ps, in parallel with more prominent bond dissociation in the borazine precursors, releasing hydrogen atoms. This phase ends at about 500 ps. From 500 ps to 1 ns the created BNf clusters undergo diffusion, and Ostwald ripening of the flakes sets in. Unsurprisingly, at an even higher temperature of 2500 K (Fig. 12c), dissociation of borazine molecules sets in even earlier at 50 ps, accompanied by an increase in the formation energy. The fragments produced by dissociation reorganize, producing B@BNc–H structures and BNc–H at 500 ps, which further reorganize and join into larger structures. At 1 ns, we can find in the products a turbostratic t-BN–H structure with a small c-BN core, and some BNf–H.

**Fig. 13:**
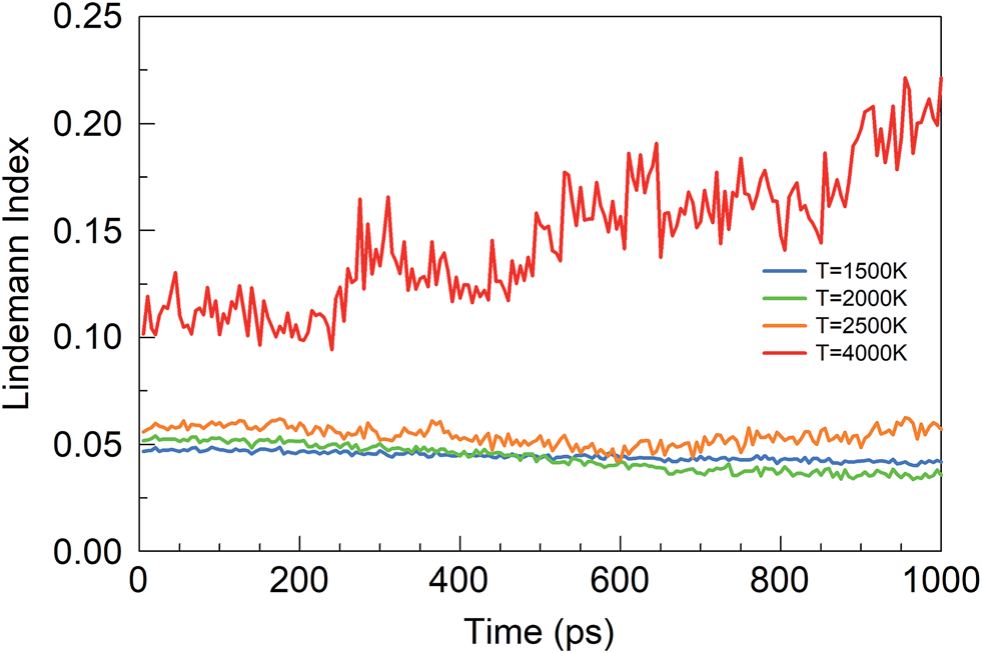
Lindemann indices for the borazine precursor system at various temperatures.

**Fig. 14:**
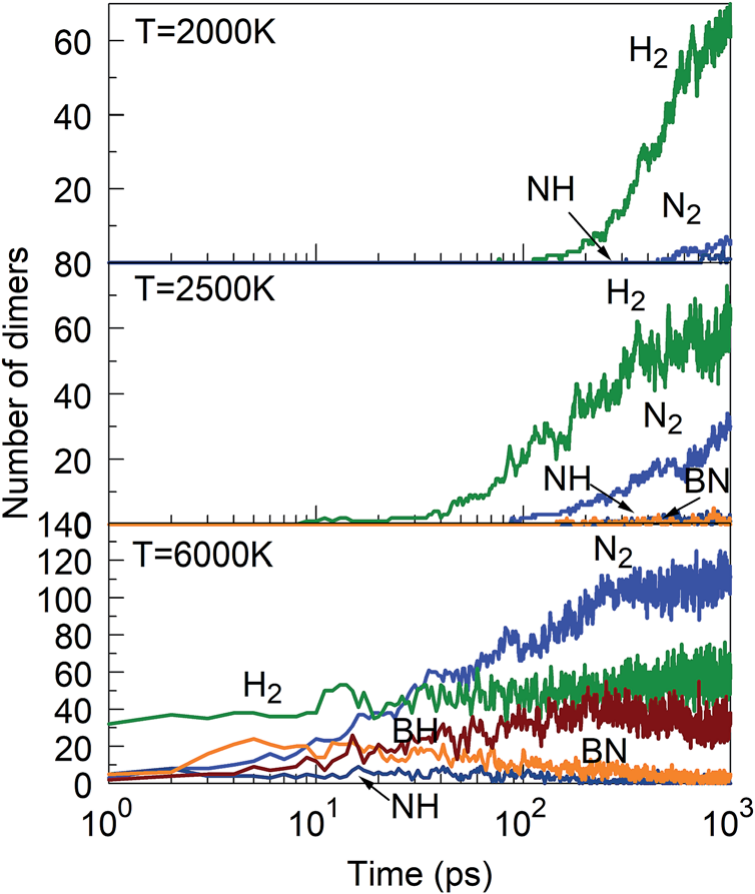
Evolution of diatomic molecules from initial mixtures of borazine molecules, for 2000, 2500 and 6000 K.

Consistent with these pictures of the transformation processes are Lindemann indices for the borazine system (Fig. 13), which show liquid cluster formation at 4000 K, and of course at 6000 K. At temperatures lower than 2500 K, the atomic motions in the created solid BNNSs are well correlated, including in a-BN–H, t-BN–H, c-BN–H and BNf–H. As seen in Fig. 14, the creation of diatomic molecules starts at about 100–200 ps for 2000 K, and is dominated by the production of H_2_ molecules formed after dissociation (bond breaking) events within the borazine molecules. The dissociation of borazine to release N and H occurs later after ∼50 ps at 2500 K. At higher temperatures, these dissociation pathways become less differentiated.

**Fig. 15:**
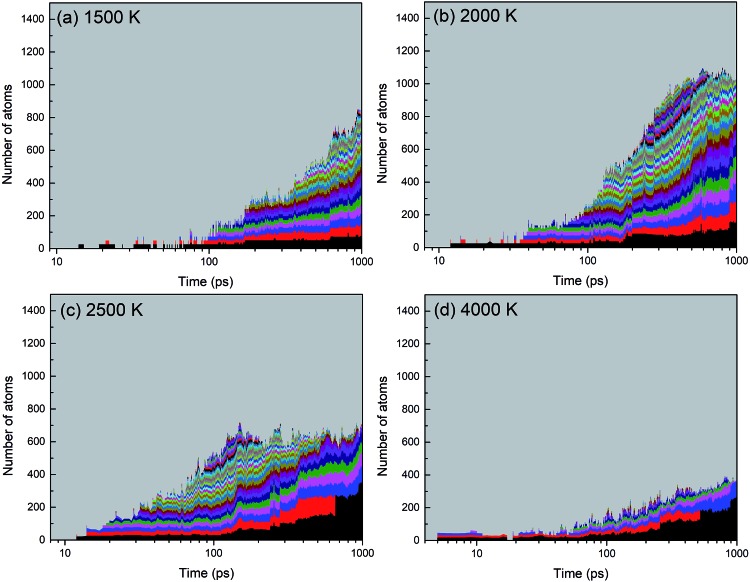
Creation and evolution of clusters from the borazine precursor systems at four different temperatures. BN structures are formed between 1500 and 2500 K, and boron clusters are formed at 4000 K and above.

**Fig. 16:**
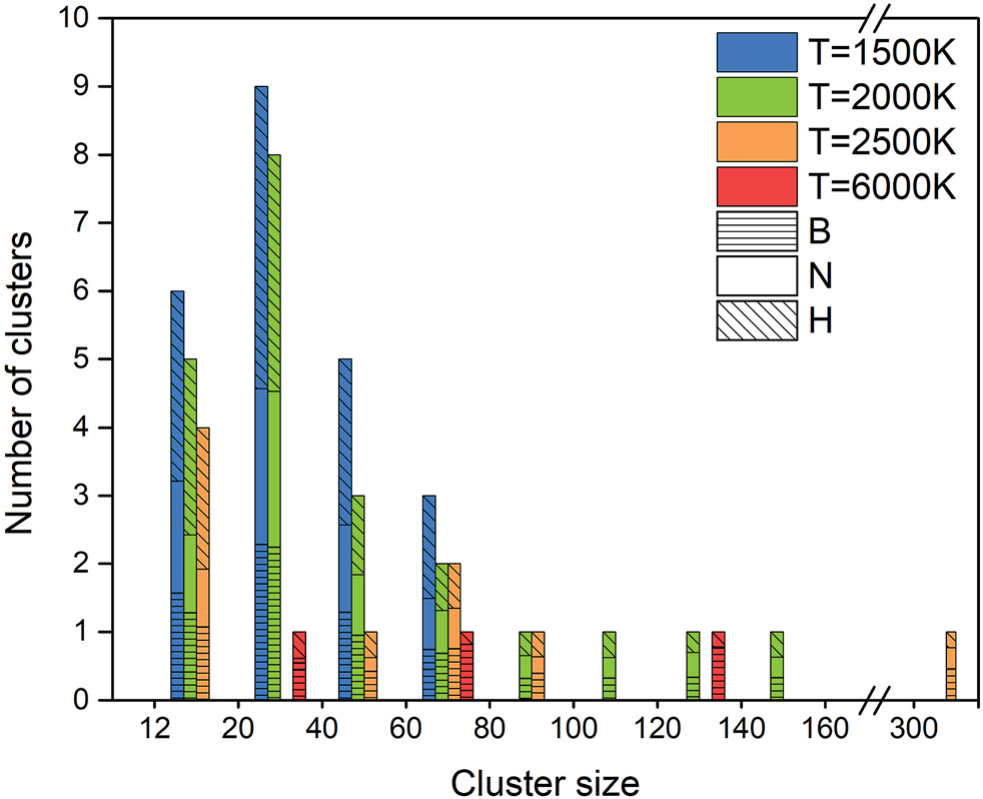
Size distribution and atomic content of clusters formed from borazine precursor systems after 1 ns for various temperatures.

Creation of clusters containing more than 12 atoms starts at about 100 ps for 1500 K, at about 40 ps for 2000 K, and at about 15 ps for 2500 K, as shown in Fig. 15. This self-assembly necessarily follows a partial or full dissociation of borazine molecules. Although a few small clusters are formed after 5 ps at 4000 K, the (at the end, mostly boron) cluster sizes remain low, containing less than one quarter of all the atoms in the system. The number of atoms captured in a cluster is largest for 2000 K, which is about 75% of all atoms, while this ratio is about 55% for 1500 K, and about 43% for 2500 K. The final cluster sizes and their atomic content at the end of the simulations are shown in Fig. 16. Thus, the biggest cluster with more than 300 atoms is formed at 2500 K, which is a cBN–aBN–H cluster, created by rearrangement of atoms after the fusion of several smaller clusters and a BN cage. Clusters of the size of about 140 atoms are formed at 2000 and 6000 K, while smaller clusters, of size below 80 atoms, are formed at 1500 K.

At this low temperature, the small clusters are dominant over the numbers at higher temperature. The atomic content of the clusters seems to be equally shared between B, N and H for bigger clusters, while smaller clusters tend towards a dominance of H.

**Fig. 17:**
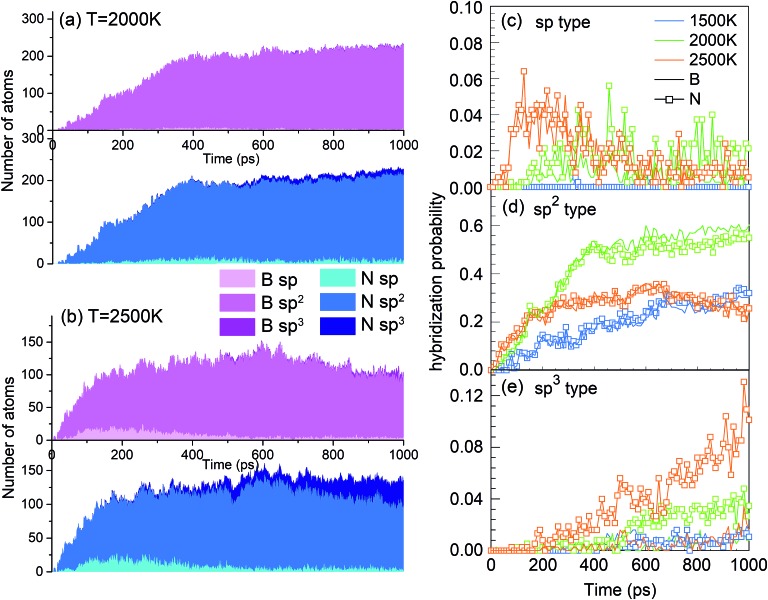
Number of sp-, sp^2^- and sp^3^-hybridized atoms for B (pink) and N (blue) at (a) 2000 K and (b) 2500 K, as functions of time for the borazine precursor system. Probabilities of formation of (c) sp, (d) sp^2^ and (e) sp^3^ B and N atoms at various temperatures, as a function of time.

A good quality check, in addition to the usually high PQS of borazine shown in Fig. 2 and 3, of the created structures over time is the count of “ideal” sp, sp^2^ and sp^3^ hybrids, shown in Fig. 17. As expected, the lack of chains as main building elements with borazine precursors results in a small number of sp hybridized atoms at all temperatures, shown for 2000 and 2500 K. A somewhat increased population of sp^3^ hybrids at longer times, especially for 2500 K is consistent with the observation of aBN–aBN–H and tetrahedral-amorphous ta-BN–H clusters. However, the overwhelming dominance of “ideal” sp^2^ atoms, both N and B, indicates the presence of either ordered structures (for example flakes) or t-BN–H structures, which may need more time to anneal into more ordered structures, like BN cages, BNNTs and flakes. The probabilities of occurrence of various hybrids in time and for various temperatures are shown in Fig. 17c–e. Thus, the probability of creating sp hybrids decreases after 200 ps, while the probability of sp^3^ hybrids increases with time, especially at 2500 K where the cBN–H and ta-BN–H structures were observed. However, the probabilities for sp^2^ hybrids are about 10 times larger than for sp^3^, and especially large at 2000 K, reaching as high as 0.6, which indicates a good “quality” of the products, in this case mostly BNf–H. This is consistent with quality assessment parameters shown in Fig. 2 and 3. We note that lower values of sp^2^ probabilities for boron in Fig. 17d are compensated by a larger number of BN hexagons, which for our observation time of 1 ns form mainly t-BN–H structures.

### 4.4 Iminoborane (HBNH)

**Fig. 18:**
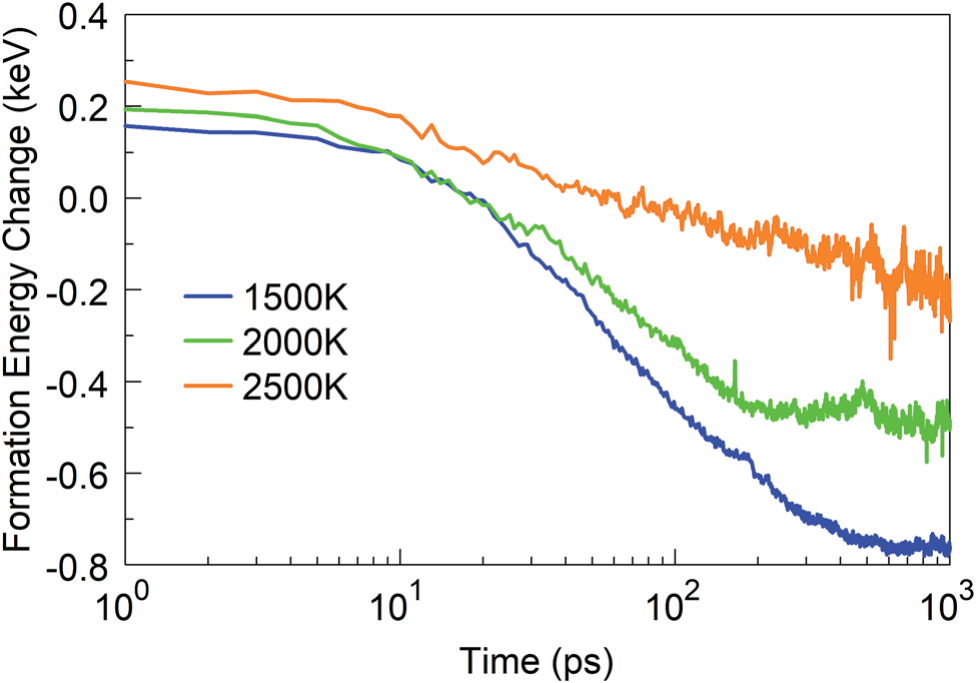
Formation energy change of the iminoborane HBNH system at various temperatures.

The stoichiometry of iminoborane, a volatile, chemically reactive compound, is the same as that of borazine, with a B : N : H ratio of 1 : 1 : 2. Having in mind the role of hydrogen in the sp^2^–sp^3^ hybridization conversion,^25^ as well as in the prevention of the closing of flake structures, this hydrogen ratio results in similar structural conversion and self-assembly features between the iminoborane precursor and the borazine precursor systems. Thus, the synthesis products at 1 ns for 1500 K are dominantly smaller flakes and amorphous structures, for 2000 K are mainly larger, joined flake structures (as shown in Movie S4 in the ESI†), and for 2500 K are amorphous turbostratic t-BN–H structures (smaller than those formed from borazine). The dimer association (which follows the dissociation of the HBNH molecules) starts at shorter times at all temperatures relative to borazine. Lindemann indices also show similar signatures of liquid boron formation at temperatures above 4000 K, and solid BNNSs with correlated atomic motions at temperatures below 2500 K. Similar notes are applicable for the atomic content of the created clusters, but the size of the largest clusters is smaller by a factor of about 2 than those in the case of borazine. However, the total number of atoms is larger in all HBNH-created clusters with more than 10 atoms, relative to borazine, by about 90% for 1500 K, about 80% for 2000 K, about 50% for 2500 K, and about 30% for 4000 K. These significantly higher numbers are a consequence of easier dissociation of the more reactive HBNH precursor, also reflected by earlier formation of dimers in comparison to borazine. Perhaps the largest difference between iminoborane and borazine is an almost two times larger number of sp^2^ hybridized atoms at 1500 K for HBNH relative to borazine, while at 2000 K, the number of sp^2^ atoms from borazine is 10% larger than from HBNH. This finding indicates that HBNH might actually represent a more effective precursor choice at low temperatures (like 1500 K) for larger ordered structures, while borazine is more effective at higher temperatures (like 2000 K). Another significant difference is the change of the formation energy in time for various temperatures, as shown in Fig. 18. This change shows a fast association and agglomeration of the radical HBNH material (for both HBNH and its dissociated species) with the HBNH precursor, while the process of agglomeration of the radicals in the case of borane is strongly conditioned by the preceding dissociation of saturated borazine molecules.

### 4.5 Atomic precursors

**Fig. 19:**
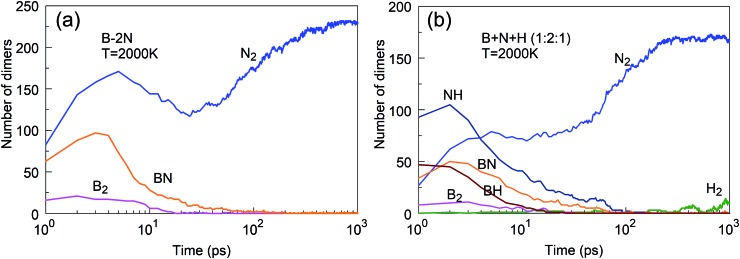
(a) Fast association of nitrogen into N_2_ suppresses synthesis of h-BN clusters; (b) fast association of NH dominates the association into N_2_ and provides reactive N feedstock to boron clusters.

We have discussed above BNNS synthesis from precursor subgroups that already have a BN chemical bond and demonstrated that they do not depend on the nitrogen feed and are more effective in the synthesis of boron nitride nanostructures. In addition, we have also studied the less productive, atomic mixture systems such as B + *x*N, *x* = 1, 2, 3 and B + N + H in ratios 1 : 1 : 1, 1 : 2 : 1, and 2 : 1 : 1, as shown in Fig. 19. The main reason that B + *x*N systems are less productive is the fast association of nitrogen atoms into N_2_ molecules, which are chemically inert and cannot effectively contribute to reactions with boron to create BN structures. The presence of hydrogen suppresses N_2_ association, by bonding of hydrogen atoms to N atoms. NH molecules are more reactive with boron than N_2_ molecules, providing in this way the needed atomic nitrogen feed. However, the use of hydrogen atoms comes at a price, as the hydrogen also binds to clusters which changes its structural (hybridization) features, as we reported earlier.^25^ Our study confirms the hypothesis put forth by Kim *et al.*^23^ that hydrogen prevents nitrogen recombination to N_2_ in the plasma synthesis of BNNTs.

### 4.6 Mechanisms of BNNT self-assembly from gas phase precursors

**Fig. 20:**
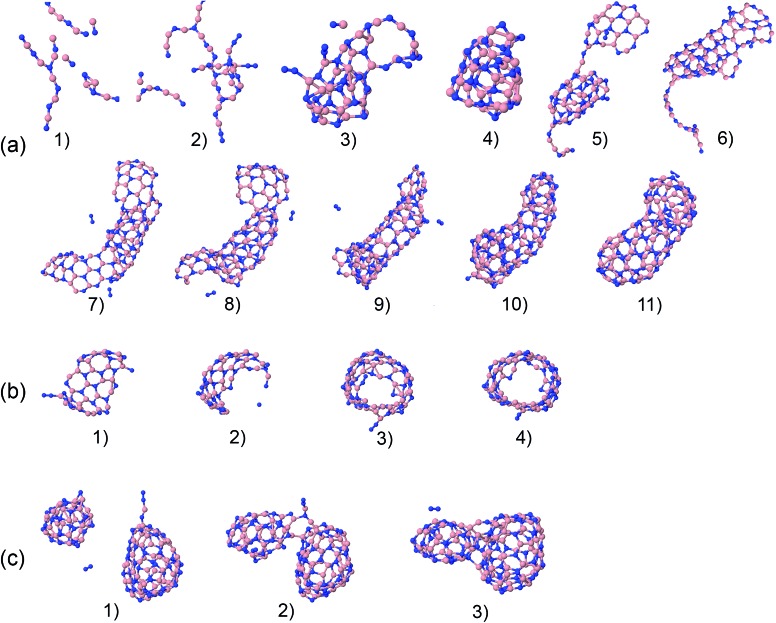
Phases of nucleation and growth of BNNT from BN precursors (a) by gradually adding up building “blocks”, (b) by folding of a BN flake, and (c) by merging of two BN cages.

Here, we focus in particular on the technologically valuable BNNT synthesis, which was observed in our simulations only from two precursor systems at a temperature of 1500 K (BN) and at 2000 K (BN, B–2N). Interestingly, we notice three scenarios that can lead to BNNT self-assembly. Fig. 20a shows the first scenario for the creation of a BNNT from a BN mixture. The BN chains branch and join (a1–a3), creating a small h-BN cluster (a4). This BN cluster connects to a BNf structure (a5), and gradually merges and reorganizes (a6). Later it “catches” another flake structure (a7), and the two structures start folding, where the diameter of the initial BN cage (a8) serves as a template for the tube diameter. Finally, through different phases of reorganization (a9–a11), the structure grows into a BNNT that is closed at both ends. We speculate on the basis of our observations that at longer times, beyond our computing time, the formed tube could “catch” more flake structures, and would grow further. Another mechanism of BNNT formation at 1500 K for the BN precursors is shown in Fig. 20b. A large BN flake is first formed by chain branching and joining in a similar fashion to that described in the previous scenario (b1), which by random folding (b2) self-connects at the edges (b3), and finally reorganizes into an open-ended BNNT (b4). A third, qualitatively different possible mechanism of BNNT growth is shown in Fig. 20c, observed at 2000 K. Self-assembled closed BN cages coalesce and reorganize into a structure which resembles a BNNT with a higher aspect ratio than a sphere. Apparently, the presence of finite-size h-BNf with reactive edges is not a necessary requirement for the BNNT self-assembly mechanism.

## 5. Conclusions

We have computationally studied the transformation and boron nitride nanostructure (BNNS) synthesis processes from a hot, high pressure gas containing various combinations of precursors B, N, and H, typically expected in plasma synthesis, to elucidate the detailed dynamics of self-assembly mechanisms into clusters and aggregates at various temperatures. Our fundamental goal is to understand the chemical dynamics and predict the optimal conditions concerning temperature and chemical composition of the precursors for controlling the synthesis process in a high temperature plasma volume at high pressure. To achieve these objectives, we have used quantum-classical molecular dynamics (QCMD) simulations based on density-functional tight-binding (DFTB) quantum chemical potential, as implemented in the DC-DFTB-K code, to study large systems containing about 1300 atomic particles in canonical NVT ensembles for 1 ns, in a temperature range from 1500 to 6000 K. By defining the “PQS” quality assessment parameter, proportional to the product of the simulated probability to create a regular, all-bond alternating BN hexagon and the probability for the formation of an sp^2^-hybridized boron atom connected to three N (or two N and one H atom in hydrogen-containing precursors) in the system, we were able to predict the four precursors that are the best candidates for actual BN nanosynthesis in plasma. The “winning” precursors are all in the molecular form and contain a B–N chemical bond. In particular, these precursors are radical BN, the closed-shell borazine (B_3_N_3_H_6_) molecule, a mix of BN radicals and H atoms in a 4 : 1 ratio, and iminoborane (radical HBNH). These precursors were able to create a significant number of BN clusters in the form of flakes, BN cages, t-BNs, and BNNTs with the smallest number of defects. When hydrogen is present in the system, it does not exert a great influence on the formation mechanism but saturates dangling bonds at the edges or on the surfaces of nascent clusters. For all studied cases, 2000 K turned out to be the most productive “winning” temperature, high enough to promote h-BN network formation yet low enough to prevent N_2_ formation, while at temperatures at or over 4000 K, the main transformation products are liquid boron clusters and gaseous N_2_ molecules. We have also studied the less productive, atomic mixture systems such as B + *x*N, *x* = 1, 2, 3 and B + N + H in ratios of 1 : 1 : 1, 1 : 2 : 1, and 2 : 1 : 1. The main reason that these systems are less productive is the fast combination of nitrogen atoms into N_2_ molecules, which have too low a reactivity to participate effectively in reactions with boron towards the creation of h-BN structures. Some of the systems that contain hydrogen are more successful in the synthesis, mainly due to the fast reaction of atomic H and N, which produces reactive NH molecules, which effectively supply the boron feed with a reactive form of nitrogen. Precursor species that already have a BN building element do not depend on the nitrogen feed and are more successful in nanosynthesis. The simulations predict the molecular, hydrogen-containing borazine and iminoborane as the most effective precursors, the former being somewhat more effective at lower temperature while borazine is more effective at higher temperature, due to its inherent chemical stability. We encourage experimentalists to employ such molecular species either as plasma additives or direct feedstock in plasma-enhanced chemical vapor deposition synthesis.

In our simulations of BNNS synthesis from small precursor species at high temperatures under high pressure we also observed the direct self-assembly of BNNTs from (a) flakes aggregating and “rolling” themselves up either by themselves or following a pre-existing BN cage diameter, or (b) alternatively directly *via* the coalescence of BN cages. These mechanisms do not require the existence of boron nanoparticles, let alone boron nitride or catalyst nanoparticles, and hence represent an alternative to the widely accepted theory of the “root growth” mechanism for BNNT formation. Rather, we find a close resemblance of the nucleation and growth mechanism to that speculated to drive the catalyst-free plasma synthesis of multi-walled carbon nanotubes.^48^

## Conflicts of interest

There are no conflicts to declare.

## Appendix A

### Glossary of the structure labels

BNoc: h-BN open cage
BNc: h-BN cage
BNcf: Joint h-BN cage and flake
B@BNc: Dominantly boron-containing cluster encapsulated inside BNc
B@BNc–H: Dominantly boron-containing cluster encapsulated inside BNc, hydrogen attached to outer atoms
BNNT: h-BN nanotube
BNf: h-BN flake
BNcf: Joint h-BN cage and flake
BNf–H: h-BN flake, hydrogen attached to edge atoms
cBN–H: Cubic BN cluster, hydrogen attached in the outer sphere
aBN: Amorphous BN cluster
aBN–H: Amorphous BN cluster, hydrogen attached to outer atoms
aB: Amorphous boron cluster, can contain small amounts of nitrogen contamination
aB–H: Amorphous boron cluster, can contain small amounts of N and H contamination. We use a concatenating “+” symbol to indicate merged clusters of different structures

## Supplementary Material

Supplementary information

Supplementary movie

Supplementary movie

Supplementary movie

Supplementary movie

Supplementary movie

Supplementary movie
