# Supplementary material for: Simulations of the synthesis of boron-nitride nanostructures in a hot, high pressure gas volume
Source: Chem Sci. 2018 Mar 19;9(15):3803–19. doi: 10.1039/c8sc00667a (PMC5939839; doi:10.1039/c8sc00667a)
Supplement: Supplementary file 1 [file SC-009-C8SC00667A-s001.pdf]

Electronic Supplementary Material (ESI) for Chemical Science.
This journal is © The Royal Society of Chemistry 2018

Electronic Supplementary Information

Simulations of synthesis of the boron-nitride nanostructures in a hot, high pressure gas volume

Predrag S. Krstic,[*a,b] Longtao Han,[a,b] Stephan Irle,[c] and Hiromi Nakai[d,e]

[a]Institute for Advanced Computational Science, Stony Brook University, Stony Brook, NY 11794-5250
[b]Department of Materials Science and Chemical Engineering, Stony Brook University, Stony Brook, NY 11794-2275
[c] Computational Sciences & Engineering Division, Oak Ridge National Laboratory, Oak Ridge TN, 37831-6493, USA
[d] Department of Chemistry and Biochemistry, School of Advanced Science and Engineering, Waseda University, Tokyo 169-8555, Japan
[e] Waseda Research Institute for Science and Engineering, Waseda University, Tokyo 169-8555, Japan

*Corresponding author, email: Predrag.Krstic@stonybrook.edu.

Table of contents

Fig. S1 Precursor - product map of the nanosynthesis products from various precursor configurations, at T=1500 K and T=2000 K.

Fig. S2 Evolution of diatomic molecules from initial mixture of BN molecules and H atoms (in ratio of 4:1) at 2000 K and 6000 K.

Fig. S3 Size distribution and atomic content of the clusters, larger than 10 atoms, formed by BN-H precursors at various temperatures, after 1 ns.

Fig. S4 Number of ideally hybridized sp, sp^2 and sp^3 atoms for B (pink) and N (blue) at (a) 2000K and (b) 4000K, as functions of time for BN-H precursor system. Probabilities of formation of (c) sp, (d) sp^2 and (e) sp^3 B and N atoms at various temperatures, as functions of time.

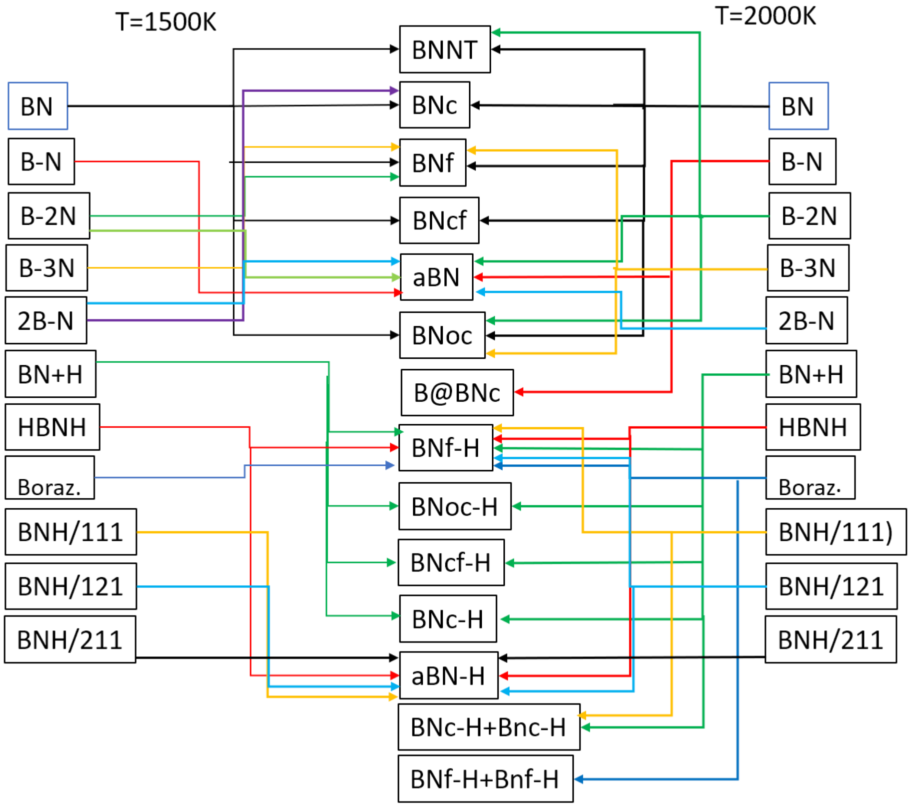

Fig. S1 Precursor - product map of the nanosynthesis products from various precursor configurations, at T=1500 K and T=2000 K.

The precursor configurations are denoted by labels specified in Table 1, and product are in same labels as shown in Fig. 1. Not all structures of Fig. 1 are shown here, because some of the structures only form at high temperatures, such as aB (amorphous boron cluster).

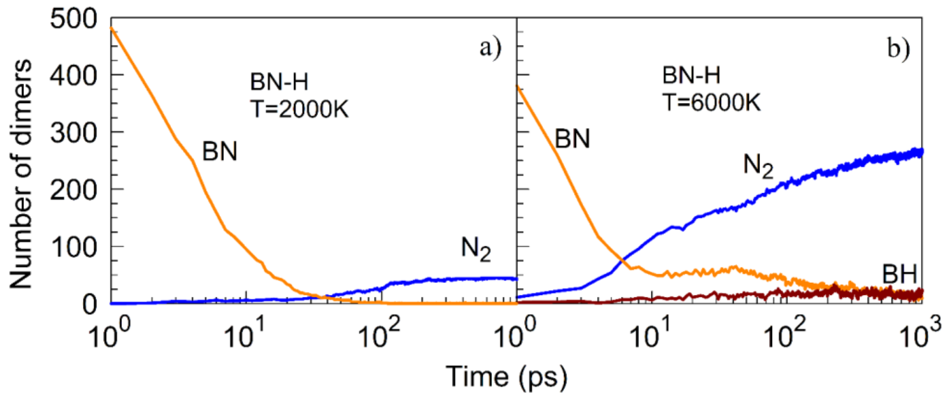

Fig. S2 Evolution of diatomic molecules in initial mixture of BN molecules and H atoms (starting in ratio of 4:1) at 2000 K and 6000 K.

Evolution of BN and N_2 molecules for BN-H precursor configuration is similar to the evolution of BN precursor configuration. At 2000 K, number of BN decreases as they are incorporated into the BNNSs. But at 6000 K, the incorporation of BN into chains and BNNSs is suppressed, and the majority of BN precursors dissociate, leading to significant association into N_2 molecules. Addition of H contribute to the formation of BH dimers at 6000 K.

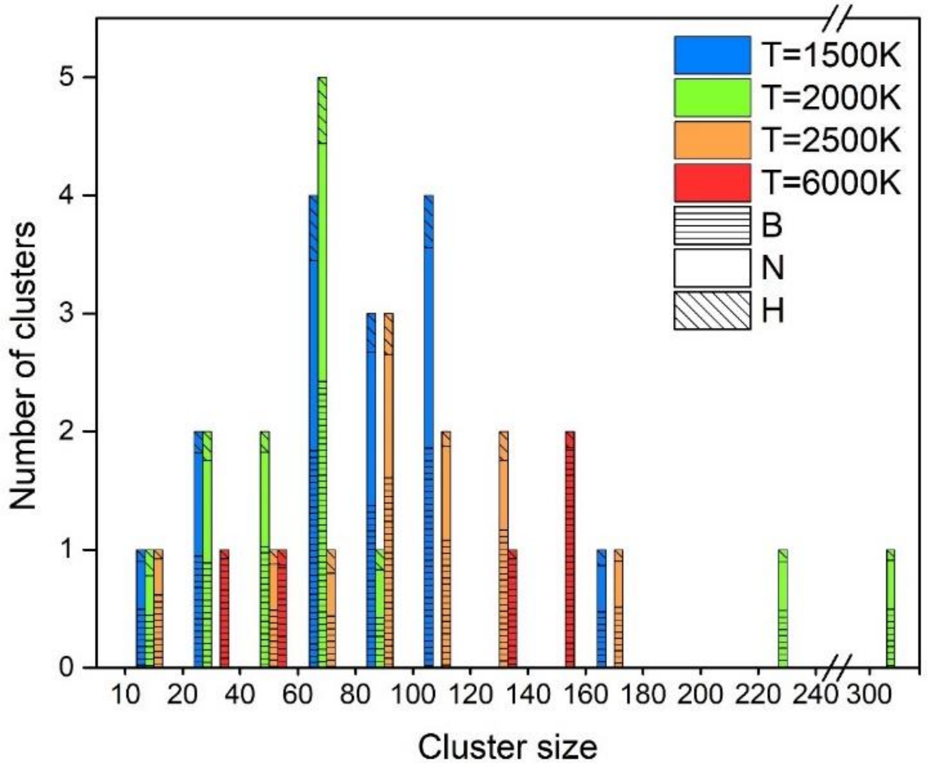

Fig. S3 Size distribution and atomic content of the clusters (larger than 10 atoms), formed by BN-H precursors at various temperatures, at t=1 ns.

Content of B in the clusters dominates over N and H and increases as the temperature increases. The presence of H suppresses agglomeration of small boron clusters into larger ones at 6000 K, as they adsorb on the external surface of boron clusters. This explains the smaller size of boron clusters formed by BN-H precursor configuration, as compared with those formed by BN precursor configuration at 6000 K.

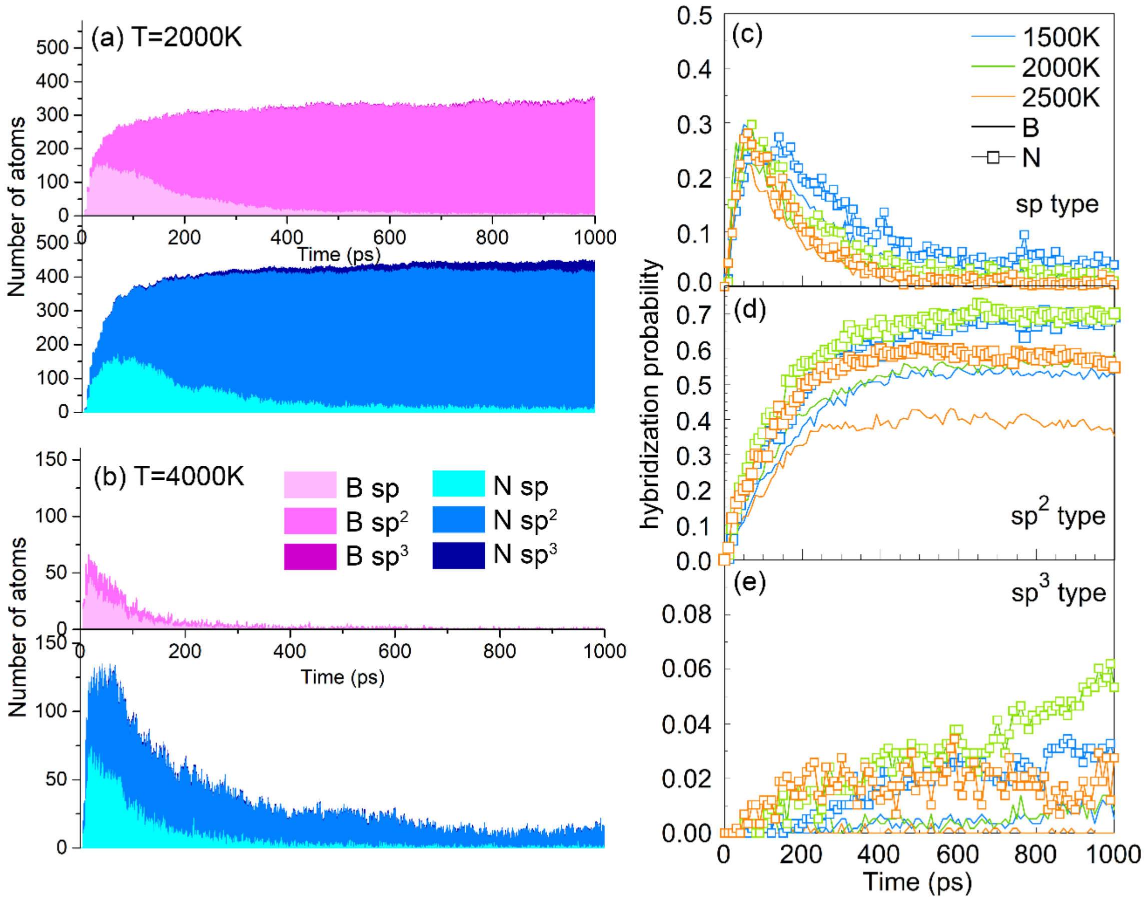

Fig. S4 Number of ideally hybridized sp, sp^2 and sp^3 atoms for B (pink) and N (blue) at (a) 2000K and (b) 4000K, as functions of time for BN-H precursor system. Probabilities of formation of (c) sp, (d) sp^2 and (e) sp^3 hybridized B and N atoms at various temperatures, as functions of time. As indicated in the legend, lines present B atoms, while symbols (squares) are for N atoms.

The content of sp^2 (in Fig. d) is by far larger than final sp^3 and sp hybrids, for both B and N atoms. Difference of N and B sp^2 hybrids is proportional to the number of defects in the structures. Initially large number of sp hybrids indicates evolution of the structures by initially forming the chains, which branch and reorganize into flake and cage structures.
